# Energy markets restructure beyond 2022 and its implications on Qatar LNG sales strategy: Business forecasting and trend analysis

**DOI:** 10.1016/j.heliyon.2024.e27682

**Published:** 2024-03-17

**Authors:** Noor Yusuf, Rajesh Govindan, Tareq Al-Ansari

**Affiliations:** College of Science and Engineering, Hamad Bin Khalifa University, Qatar Foundation, Doha, Qatar

**Keywords:** LNG, Time series model, Exponential smoothing, And natural gas pricing

## Abstract

The emergence of new suppliers and energy resources has reshaped the energy market in terms of contractual structures and pricing systems. The market shifts were accelerated in response to the latest Russian-Ukraine crisis, impacting natural gas supply chains from financing projects to contracting volumes. The increased demand for liquified natural gas volumes intensified the need to switch from long-term oil-indexed contracts to short-term gas-indexed contracts. Those shifts were anticipated to influence the selling strategies for the expected added 49 MTPA of Qatari LNG, wherein increasing the share of spot selling would be reflected in higher economic performance. This study used forecasted prices to investigate potential Qatari LNG selling strategies. Initially, projections of the most dominant pricing systems used for pricing Qatari LNG (i.e., brent, Henry Hub, Title Transfer Facility, and Japan Korea Marker) were estimated between 2023 and 2040. While Qatar has been relying on long-term oil-indexed contracts, the second step estimated annual LNG revenues under different combinations of selling strategies (i.e., long-term and spot sales). Finally, the influence of varying brent slopes on the estimated revenues was measured. Due to data limitations and non-stationarity, the double exponential smoothing model was selected among the different tested models. Considering current market dynamics, forecasts of the double exponential smoothing model showed an upward price trend until 2040. An annual average increase of 1.24% for the studied pricing systems was reported. Reducing the share of long-term brent-indexed contracts to 70% and dedicating the remaining 30% of volumes to spot sales yielded the highest premiums for revenue estimates. An average annual revenue of $62 bn was reported for the 70/30 strategy, around 6% higher than the 100% brent-indexed contracts strategy. The findings revealed that diversifying the selling approach and introducing spot sales can enhance revenues. From the buyers’ perspective, the outcomes support policymakers in understanding the implications of escalated prices driven by a lack of liquidity investments.

## 1 Introduction

In the last few years, a substantial global movement has been recognised to minimise fossil fuel consumption. Concerns about the unsustainability of fossil fuels, global climate change, environmental implications, and volatility of energy commodity prices drove the shift. On the contrary, the interest in natural gas as a cleaner fossil-based energy resource has emerged in several national energy roadmaps. Supported by net-zero aspirations, industry experts project more reliance on natural gas as a bridge fuel to renewables [[1], [2], [3]]. The EIA predicts an expansion of natural gas consumption by more than 40% in 2050 relative to 2018 [4]. Compared to other fossil fuels, burning natural gas emits less CO_2_ to generate the same energy content [5]. For example, combusting natural gas emits 65% less CO_2_ than coal in power generation applications [6]. Consequently, countries like China and India promoted coal-to-gas policies as part of their climate mitigation targets [7].

Especially with the evolution of different natural gas monetisation options, liquified natural gas (LNG) has gained attention for economical transportation to distant markets [[8], [9]]. The LNG supply chain consists of 4 main sections: (i) exploration and production, (ii) liquefaction, transportation, and storage, (iii) regasification at receiving terminals, (iv) and distribution to domestic markets [10]. In an LNG supply chain, the producer is responsible for financing and managing the production and liquefaction infrastructure, whilst the consumer oversees funding and managing the regasification infrastructure and supplying natural gas to local consumers. Amongst the different parts of the LNG supply chain, liquefaction is the most capital-intensive, with a capital breakdown of 42% [11]. In addition to the high costs, liquefaction plants typically take around 10 years to fully develop, which includes 6 years of planning and 4 years of construction.

The high capital and operating costs of liquefaction projects have classically imposed the need for long-term contracts (LTCs) for more than 20 years to secure sales. However, since the U.S. shale gas revolution, the market dynamics have shifted towards shorter-term contracts and spot selling. The availability of high uncontracted volumes decreased the willingness of LNG consumers to commit to LTCs and strict duration clauses. This, in turn, influenced the financing and decision-making process of new planned LNG projects. Additionally, several recent shocks triggered contractual structures and LNG projects in the pre-final investment decision-making phase. The shocks include the collapse of oil prices in March 2020 due to the COVID-19 pandemic lockdown and natural gas price spikes driven by the Russian-Ukraine crisis in 2022. Amongst several affected projects, Australian LNG projects were delayed due to financing challenges. Furthermore, the focus on Europe as a more profitable market with higher incentives was brought to the exporters’ attention after the Russian sanctions driven by the need for LNG volumes to substitute pipeline gas.

Supported by the low natural gas supply and liquefaction costs, the operation of Qatari LNG plans remained unaffected. More recently, Qatar announced expanding its production capacity from 77 to 126 MTPA by 2027 to meet the growing demand. On the contrary, the spike in spot energy commodity prices significantly restructured the global energy markets. Various buyers reconsidered signing LNG sales and purchases agreements (SPAs) to hedge against price volatility. In terms of pricing systems, the Title Transfer Facility pricing system (TTF) has been a price setter in the global natural gas market in 2022 [12,13]. The surge in European demand for LNG to replace Russian pipeline gas motivated sellers to divert non-contracted spot cargoes from Asia to Europe for higher premiums. In parallel, price-sensitive Asian markets showed higher vulnerabilities to the price surge imposed by the Russian-Ukraine crisis. For example, high natural gas costs forced roughly 25 GW of India's gas-fired power capacity to shut down, jeopardising natural gas infrastructure development based on a pricing forecast of less than $10/MMBtu [14]. In terms of selling strategies, the latest market changes increased the appetite of the largest LNG suppliers to diversify their selling portfolio by targeting high-premium European markets. Qatar started acquiescing regasification capacities at European receiving terminals to diversify its spot market share away from Asian markets. Additionally, the surge in spot prices pushed buyers to rely on spot cargo to return to LTCs to hedge against volatile spot prices and secure LNG supplies.

**Fig. 1:**
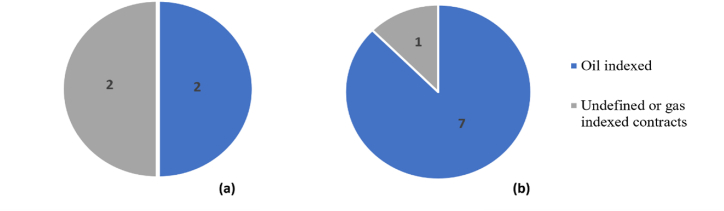
Proportion of oil-indexed to undefined or gas-indexed LNG contracts signed in years 2022 (a) and 2021 (b).

Qatar's LNG, crude oil, and petroleum exports account for a significant share of the government's total earnings. Hydrocarbon revenue provided approximately 37% of Qatar's GDP in 2021, a 9% rise over 2020 [15,16]. In terms of LNG sales, Qatar has a strategy of dedicating 90–95% of its LNG production capacity to LTCs with strict duration clauses to hedge against risks. However, with changes in contractual structures and oversupplied volumes from competitive suppliers, Qatar was highly challenged to secure LTCs. The saturated market demanded multiple changes to be considered to resign new SPAs. This includes shifting from oil-indexed to gas-indexed pricing and introducing flexibility to contracted volumes and delivery schedules according to market conditions. In 2021 and 2022, Qatar signed 12 new LTCs with buyers as part of the LNG expansion plans (Fig. 1). However, the expiry of several SPAs and the challenges in securing new SPAs for the added capacities have concerned decision-makers. From a buyers' perspective, energy import security has been critical for regions with limited fossil fuels, such as Europe. Hence, significant research efforts have been recognised in the literature to assess and optimise the energy portfolios for import and domestic utilisation purposes [[17], [18], [19], [20]]. Moreover, several studies reported analytical frameworks on the LNG importation portfolio to investigate the optimal strategies [[21], [22], [23], [24]]. For producing companies, early market assessments facilitate deciding and planning production capacities, selling portfolios, and delivery plans [[25], [26], [27]]. The uncertainties associated with energy markets and the increased competition motivated researchers to investigate and assess optimal LNG export strategies [[28], [29], [30]]. In the literature, most of the natural gas export portfolio management studies focused on assessing the diversification of existing portfolios using different metrics, including the Herfindahl-Hirschmann index and Shannon-Wiener concentration index [24,29,[31], [32], [33]]. However, the literature did not empirically examine the considerations of changes in pricing and contractual structures. The work presented herein highlights an integrated predictive-prescriptive framework to guide natural gas decision-makers on long-term strategic LNG portfolio planning. The analysis focused on Qatar as a case study, where liquified natural gas dominates the export portfolio through answering the following research questions:

(1) How can historical data on past crises and their effects on natural gas markets be used to develop robust forecasting models for predicting future price trends and market dynamics?
(2) What is the implication of varying global demand and prices on the profitability of existing LNG projects?
(3) What are the implications of long-term contracts versus spot market transactions for Qatar's natural gas exports during crises, and how do pricing mechanisms differ between these two models?
(4) What potential selling strategies could be utilised by Qatar for the added uncontracted volumes?

Compared to other studies in the literature, the outcomes of this work support investment decision-makers and policymakers with a crucial understanding of different factors influencing the economic sustainability of the Qatari LNG industry.

The rest of this study is structured as follows: section 2 highlights the research progress on natural gas/oil markets. Section 3 discusses the methodological approach. Section 4 reports the empirical results with a detailed discussion of the results5 and benchmarks the price forecasts presented in this work with forecasts by third-party agencies. Section 5 illustrates the policy implications of the results. Lastly, section 6 highlights the conclusions and main findings.

## 2 Literature review

### 2.1 Empirical studies

**Fig. 2:**
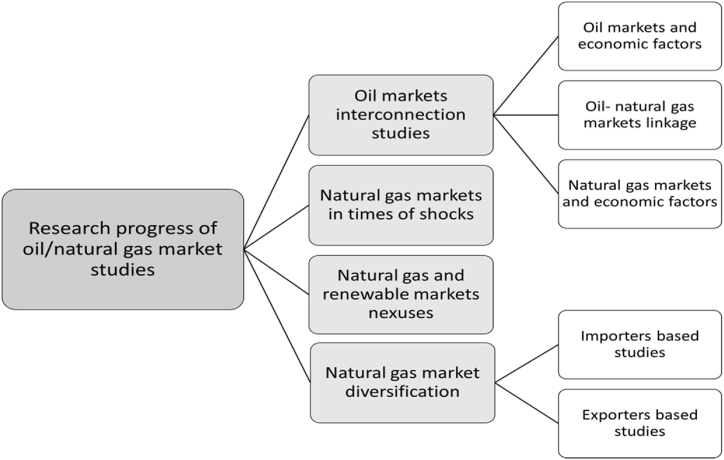
Overview of research progress on oil/natural gas market studies.

This section summarises four categories of literature correlated with our work. First, the interconnection between oil/natural gas markets and economic factors such as the stock market was discussed. Second, assessments of the latest COVID-19 pandemic and the Russian-Ukraine conflict on natural gas prices were addressed. Third, natural gas market nexuses with renewable energy markets and sustainability targets were highlighted. Finally, natural gas market diversification studies in the strategic decision-making domain were discussed. Fig. 2 summarises the different areas of reviewed studies in oil and natural gas markets.

For the first stand, Hamilton [34] suggested the strong correlation between oil price changes and national product growth in the US between 1948 and 1972. Since then, the relationship between oil prices and different sectors of the economy has been of significant interest to scholars, traders, and policymakers. In the literature, further theoretical and empirical studies addressed the influence of oil price shocks on economic activities [[35], [36], [37], [38], [39]]. Quantitative tests such as Granger causality, quantile regression, couple functions, generalised autoregressive conditional heteroskedasticity (GARCH), and vector autoregression have been common to assess the co-movement and test for the effect of oil price change [40,41]. Amongst the different co-movement assessments, the linkage between oil price change and the stock market gained significant attention with the hypothesis that stock market performance reflects a healthy economy [[42], [43], [44], [45], [46], [47], [48], [49], [50]]. In the reviewed studies, it was observed that the effect of oil price changes on the stock market varies depending on the methodology, time horizon, and the distinctive economic structure of the assessed country. The empirical studies on oil price linkage with stock market prices/returns were extensively discussed and clustered based on findings (i.e., significant positive impact, significant negative impact, and insignificant impact) as reported in Ghosh & Kanjilal [44] and Mensi et al. [51].

Furthermore, the expansion in natural gas consumption motivated scholars to assess the impact of natural gas price fluctuations on energy policy and the stock market [[51], [52], [53]]. Natural gas and crude oil have been used interchangeably in industry, and the commodities are known to complement each other in power generation. Given this argument, the influence of natural gas price changes on the stock market can mimic the influence of oil price changes on the stock market. Earlier assessments revealed that natural gas and crude oil markets are highly dependent [[54], [55], [56], [57]]. This is due to the maturity of the oil-linked indexations used for pricing natural gas contracted volumes [58]. However, the latter argument indicates that the oil market leads the natural gas market [59]. A study by Ramberg et al. [60] on the interconnectedness between oil and natural gas markets concluded a weak connection between the oil and natural gas markets. On the contrary, the enhanced maturity of the natural gas pricing systems motivated distinct assessments of the spillover effect between the natural gas market and stock market prices/returns [51,52,59]. An assessment by Acaravci et al. [61] revealed that the natural gas market indirectly impacts the stock market. The results of the study concluded that increased natural gas prices influence manufacturing growth, which will be extended to influence stock market prices. Of late, the linkage between natural gas and different sectoral and aggregate stock market levels, such as S&P 500 returns [31], Qatar's stock market returns [62], China's stock market [53], US-UK-Europe stock markets [31], and India's stock market [59] were assessed. Overall, it was concluded that only a few studies in the literature covered the relationship between natural gas and stock markets.

Natural gas has been a key commodity influencing multiple economies, including rich gas-producing countries. Identifying the transmitted impact of natural gas price shocks has been a key priority for investors and portfolio managers to provide insights on portfolio diversification and risk minimisation strategies [63]. In recent literature, several studies have focused on assessing the interrelationship between natural gas market prices and economic activities during the latest COVID-19 pandemic and the Russian-Ukraine conflict. In the case of asymmetric link assessment between COVID-19 and fossil energy markets, studies were clustered based on three groups. The first group assessed the interlink between the COVID-19 pandemic and energy firms [[64], [65], [66], [67]]. The second group focused on the impact of COVID-19 on energy markets [[68], [69], [70]]. The third group empirically examined the link between energy and stock markets during the COVID-19 pandemic [[71], [72], [73], [74]]. For natural gas markets, an empirical assessment by Cieślik [75] evaluated the impact of natural gas consumption on the domestic market in a city in Poland during the pandemic. The results revealed that the decline in natural gas consumption passed on risk to the financial standing of suppliers and consumers. Like other economies, the drop in consumption created uncertainty in economic development and companies' revenue growth. The impact was extended to a drop in companies' share prices listed on the Poland Warsaw Stock Exchange, as investigated by Bieszk-Stolorz [76] for the first quarter of 2020. Abadie [77] reported a stochastic diffusion model for forecasting Spanish daily quotes using the end of 2019 as a starting point in the context of modelling wholesale natural gas markets during shocks. Forecasted prices were reported to be between 47 and 62% less than the actual values in the first wave of the pandemic between April and June 2020. While the COVID-19 pandemic was a demand disruption concern from producers' perspective, the shortage of natural gas supply due to Russian sanctions has been a consumer problem. European importers' surge in demand for spot LNG cargos impacted the global LNG market, including developing economies. The latest studies in the literature reported the economic impact of the Russian-Ukraine crisis on the energy markets of the US, UK, Canada, and Europe [[77], [78], [79], [80], [81], [82], [83]]. Covering different shocks between January 2015 and March 2022, Uribe et al. [84] investigated the transmission of natural gas shocks to electricity prices in 21 European markets. Based on quantile slopes as indicators, the results revealed that Denmark and Finland are the most vulnerable when the natural gas market is distressed. Portugal and Spain were concluded to be the most resilient to natural gas shocks. Overall, the investigations in the literature focused on importers' vulnerabilities and global energy market price surges. To the authors’ knowledge, no study empirically reported the impact of the crisis on the selling strategy of natural gas exporting countries in times of supply disruptions.

For the third stand, the nexus between natural gas markets and renewable energy markets and sustainability targets are discussed. With the emergence of energy market shocks in the last decade, shifting to and financing renewables has been questioned. In the literature, studies assessing renewables markets were divided into two groups. The first group of studies primarily analysed the connectedness between natural gas and renewable energy markets, focusing on event-based approaches [[85], [86], [87], [88], [89], [90], [91]]. Empirical tools such as quantile-based regression, quantile-on-quantile, and causality-in-quantiles were present in the studies. The study outcomes suggested a noticeable transmission of natural gas markets' volatility to renewable energy tokens. This reflects the novel link between renewable energy and geopolitical conflict resolution. On the other hand, consumer behaviour plays a crucial role in accelerating the transition to renewables. The second group of studies adds to the theory of planned behaviour, wherein the public perception of renewables is identified based on variables such as environmental knowledge, environmental concern, and beliefs [[92], [93], [94], [95], [96], [97]]. Based on empirically assessed collected data from questionnaires, outcomes emphasised that consumers' behaviour is driven by their attitude. It was revealed that increased awareness of environmental concerns is a driving force to purchase intention. Such comprehensive research on public attitudes towards renewables provides decision-makers and authorities with insights into the requirements to enhance the public's adoption of renewables.

The fourth stand highlights studies on diversifying natural gas portfolios in response to the implications of natural gas market variabilities on energy security. In the literature, studies addressed the natural gas and LNG trade dynamics and the ability of suppliers to secure long-term contracts as an indicator of demand stability [22,24,[98], [99], [100]]. Given the considerable share of income obtained from energy commodities exports in national GDPs, demand turbulence significantly impacts national economies. A few studies highlighted the importance of export diversification for exporting countries and regions using measures of market concentration tools, such as the Herfindahl-Hirschmann index (HHI) and Shannon-Wiener concentration index (SWI), to calculate and classify the level of LNG diversification. The investigated studies focused on exporting countries/regions such as Russia [29,101], Turkmenistan [32], Qatar [29], Australia [29], the U.S. [29], and Malaysia [29], and Asia [24,33]. Fundamentally, HHI and SWI are competent, and there is no compelling rationale to prefer one. However, SWI prioritises the influence of smaller market players, whereas HHI prioritises larger buyers [102,103].

For exporting countries, it is worth mentioning that regional natural gas pricing systems must be considered when optimising an export market portfolio. Unlike oil markets, natural gas markets are segmented into regional markets with localised pricing mechanisms. Two main pricing systems dominate the natural gas markets: (1) oil-indexed pricing using brent crude prices and Japan Crude Cocktail prices (JCC), and (2) gas-indexed pricing using the U.S. Henry Hub (HH), Dutch Title Transfer Facility (TTF), UK National Balance Point (NBP), and Platts Japan Korea Marker (JKM). A concrete understanding of the pricing systems adds to building a robust portfolio to maximise profitability and/or minimise risks. Time series analysis has been a widely used empirical approach to understand the factors driving price behaviour [[104], [105], [106]], the interconnection between the pricing systems [[107], [108], [109]], and to predict future values [[110], [111], [112]]. Overall, investigating the pricing systems for reshaping policies or liberalising local pricing hubs from the perspective of importers has been a dominant theme in former studies.

### 2.2 Theoretical background

Screening the literature revealed that the dominant theme of studies was concerned with interlink assessments between energy markets and different economic factors. Additionally, with increased fluctuations in energy commodities demand, more recent studies explored energy security from the perspective of oil and gas exporters [113,114]. Few studies reported natural gas portfolio diversification studies from buyers' perspectives. However, the latter studies did not investigate forecasting different pricing systems nor investigated exporters’ perspectives. The shifts in the contractual structures, pricing systems, and the appetite for LNG imports in Europe are reshaping the energy market. Hence, it is crucial to investigate the influence of the latest market dynamics on Qatari LNG revenues based on different exporting scenarios. In the literature, Günther & Nissen [115] studied the daily effects of the HH price on U.S. LNG exports and gas flows in Western Europe at the beginning of the 2030s using the global gas market model WEGA. The utilised model reflects a regional focus on natural gas flow within domestic markets. In contrast, LNG flows are considered an external parameter that does not reflect the accurate scenario of fundamental market dynamics. In the case of Qatar, a major exporting country with a high reliance on oil-indexed LTCs, investigating the potential impact of the latest trend on the expansion plans is insightful for decision-making. A few studies in the literature addressed the future Qatari LNG export outlook and its competitiveness in the international markets at different time periods. Factors such as the influence of LNG development on the Qatari economy, the U.S. shale gas revolution, and the impact of the COVID-19 pandemic on the expansion plans were evaluated [28,[116], [117], [118]]. A recent study by Al-Breiki & Bicer [119] suggested short=to medium-term solutions to be considered by Qatar for tackling the European natural gas shortage after the Russian sanctions. To seize market opportunities, the authors proposed the domestic use of renewables for power generation and exporting natural gas to Europe. However, the study focused solely on the technical aspects of the solutions without addressing economic perspectives.

To date, no other studies in the literature have focused on the economic aspects and the impact of different selling strategies on Qatari LNG revenues. In this work, the influence of the latest energy market restructuring on the long-term Qatari LNG annual revenues for LNG volumes sold in Asian and European markets is investigated. First, annual natural gas and oil prices are projected based on observations of the most utilised pricing systems in the Qatari sales and purchase agreements. Time-series exponential smoothing models are considered for forecasting brent crude, Henry Hub, and Title Transfer Facility prices in a business-as-usual scenario until 2040. Second, assuming Qatar will sustain a 75/25 market share in Asia and Europe and utilising real contracts data, a scenario-based analysis is conducted to estimate the annual LNG revenues based on different selling strategies.

## 3 Data and methods

### 3.1 Available data

**Fig. 3:**
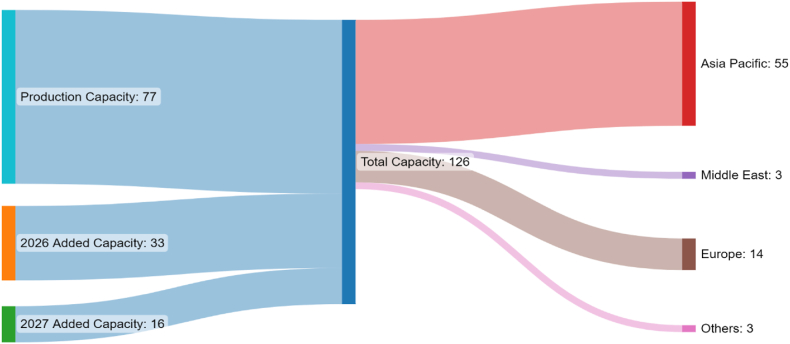
Contracted Qatari LNG volumes (MTPA) to different regional markets in 2027.

With added LNG volumes of 49 MTPA to the market by 2026 and the expiration of several long-term contracts, Qatar remains challenged in terms of securing LNG sales to sustain the business. Qatar's LNG marketing strategy has traditionally relied heavily on LTCs to secure sales and hedge against market vulnerabilities. With Asia Pacific being the main market, JCC-indexed contracts were the most dominant. In the last few years, Qatar began diversifying its LNG pricing strategy using different oil and gas indexations (i.e., HH, JKM, TTF, and brent) to reduce the risks of relying on a single pricing system. Brent indexation has been the most significant in the recently signed contract with different slopes (i.e., degree of indexation) used for different buyers. During the lifetime of the contract and the initial clauses, the slope is renegotiated every 5–10 years based on market conditions. Fig. 3 illustrates the up-to-date annual LNG contracted volumes with different buyers by 2027. All stated LNG volumes are exported from Qatar-based plants in Ras Laffan: Qatargas I, III, and IV, and Ras Laffan RasGas II T1, T2, and T3. In addition to domestically produced volumes, added capacities are anticipated from international projects in the future. This includes volumes from the Golden Pass LNG project in the US, a joint venture between Qatar Energy and ExxonMobil affiliates that will become operational in 2027. Yet, no contracted volumes have been announced for the project.

Given the lack of transparency in the LNG industry and the confidentiality of contracts, it is uncommon to publicise contract clauses. Where this has been done to comply with government/regulatory requirements in some cases, obtaining oil slope data of some contracts was possible. Based on the following available contract data, a weighted average brent-indexation of 11.79% was calculated, as represented in Table 1. Although the total contracted volumes are around 27.5% of the up-to-date contracted volumes in 2026, the calculated weighted slope is within the acceptable range used in practice (10–16%). It is considered a valid assumption to be applied when estimating the revenues of overall LNG sales to different international markets. A slope of 115% is used for HH-indexation based on published reports assuming that the Qatari LNG is competitive with the U.S. Sabine Pass LNG exports [120,121].

**Table 1:** Calculated Weighted Average brent slope based on published slope values.

Contract	Volume (MTPA)	Slope
Sinopec (China)	2	10.19%
Pakistan State Oil Company (Pakistan)	3.75	13.37%
Pakistan State Oil Company (Pakistan)	3	10.20%
Petronet (India)	8.5	12.67%
KPC (Kuwait)	3	10%
Total volume/weighted average	20.25	11.79%

**Fig. 4:**
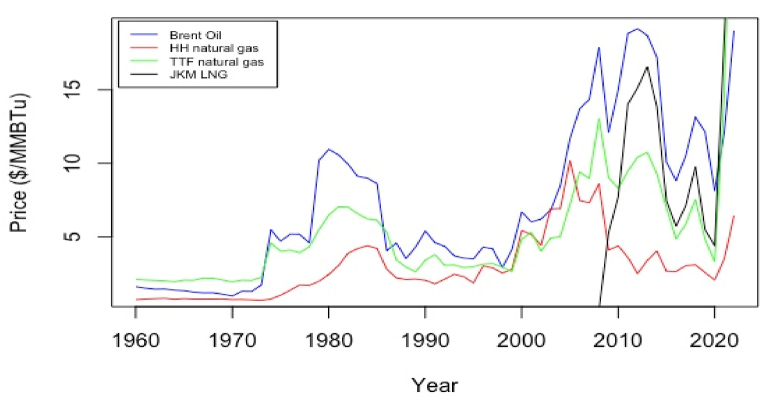
Average annual prices for brent, HH, TTF, and JKM pricing systems.

In addition to brent indexation, other pricing systems were observed in LNG sales, such as HH for American markets, TTF for European sales, NBP for LNG sales in the UK, and JCC and JKM for pricing volumes to the Asia Pacific markets. In this analysis, we estimate the revenues for LNG sales in different regional markets by considering the most prominent pricing systems used per region: brent, HH, TTF, and JKM. Fig. 4 illustrates the historical prices used as input to forecast future values. Forecasted values are the main input for estimating Qatari LNG revenues in regional markets. For the empirical analysis, two main computational software were used: R software for statistical analysis and Microsoft Excel for calculating the revenues based on the following assumptions:

• Historical real prices were used to forecast future values while accounting for inflation. All calculated revenues are given in present value.
• The share of the sales in the Asian market was anticipated to remain up to 75% in the long term, driven by demand growth in China and India. At the same time, the remaining market share was allocated to European markets.
• A brent slope of 11.79% and an HH slope of 115% were considered.
• The revenues were calculated based on Equation (1), wherein b is a Qatar-based liquefaction cost of $1.69/MMBtu.
• All sales were assumed to be delivered ex-ship. Hence, the shipping costs were not considered.

### 3.2 Methodological framework

Historical prices and contract data were primarily used to estimate regional LNG sales based on different combined sale strategies and pricing scenarios. A three-step methodology was considered to estimate the revenues: (1) predictive modelling for forecasting future prices, (2) scenario-based analysis for revenue estimation, and (3) sensitivity analysis around the scenarios. The following subsections detail the forecasting models and scenarios used for revenue estimation.

#### 3.2.1 Predictive modelling for price forecasting

Various machine learning and artificial intelligence models have been used for forecasting future data based on historical time series, such as neural networks and autoregressive integrated moving average (ARIMA) [122,123]. Observations from the literature reveal that the availability of daily datasets supported the flexibility in investigating different models for forecasting short-term data (i.e., daily and weekly) [124,125]. Generally, the optimal model selection is subject to the sample size, the predicted period (i.e., short, medium, or long), the amount of randomness in the data, and the number of parameters to be estimated by the model. The requirement for a larger sample size is directly proportional to the number of estimated parameters and the amount of noise in the data set [126]. For nonstationary time series data, exponential smoothing models proved their efficiency for forecasting future values by considering different factors. The exponential smoothening algorithm is a forecasting technique that uses an exponential window function to smooth time series data and forecast new values. Unlike the simple moving average method, exponential functions apply weights that decrease exponentially over time. Greater weights are placed on the more recent values, while the importance of older values exponentially decreases with time.

Exponential smoothening models (ES) are classified based on the combinations of error, trend and seasonality terms. ES models include a simple exponential smoothing model (SES) with no trend and seasonality terms, a double exponential smoothing model (DES) with a trend term, and a triple exponential smoothing model (TES) to handle time series data with trend and seasonality. In this analysis, additive DES, also known as Holt's method, was considered to estimate the trend in the data and the level. For s_1_ = x_1_, b_1_ = x_1_-x_0_, and t > 1, Equations (1), (2) describe the mathematical representation of the DES model.

(1) $$s_{t}=\alpha x_{t}+\left(1-\alpha \right)\left(s_{t-1}+b_{t-1}\right)$$

(2) $$b_{t}=\beta \left(s_{t}-s_{t-1}\right)+\left(1-\beta \right)b_{t-1}$$

where b_t_ is the trend factor at time t (i.e., the best estimate of trend) and β is a trend smoothing constant between 0 and 1*.* In a DES model, α and β are preliminary specified by the model user.

#### 3.2.2 Scenarios for revenue estimation

**Fig. 5:**
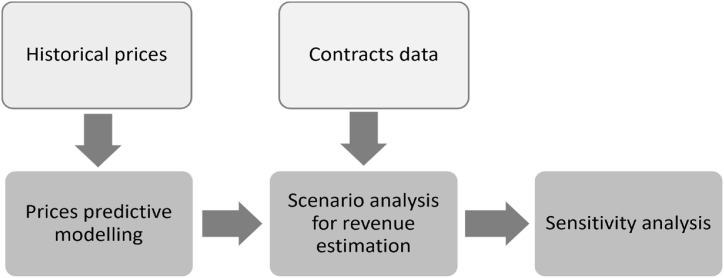
The methodological framework used to estimate the annual Qatari LNG revenues.

Due to the confidentiality of the pricing formulas used in LNG SPAs, four scenarios were assumed for estimating contracted and spot LNG revenues in regional markets. Two major contractual structures were considered in the scenarios. First, LNG volumes are fully contracted to different regional markets. Second, 70% of the Qatari LNG volumes are contracted, while the remaining volumes are sold in the spot market or based on short-term gas-indexed agreements. Brent, TTF, and HH pricing systems were considered for contracted LNG volumes. HH, TTF, and JKM were assumed to estimate spot sales in different regional markets based on the allocated shares. While brent has been the most dominant pricing system for Qatari contracts, the remaining pricing systems have acquired an interest in the latest signed Qatari LNG SPAs. Table 2 summarises the addressed scenarios in terms of selling strategy, pricing mechanism, baseline slope, and uncontracted volumes market share. Scenario A.1 considers the current selling strategy adopted by Qatar. Scenario A.2 estimates the influence of shifting to HH indexation for newly signed LTCs on the revenues. Scenarios B.1 and B.2 evaluate the economic attractiveness of reducing the current LTC strategy from almost 100% to less than 70%. The latter two scenarios represent the worst possible strategy for Qatar in case of failing to secure long-term SPAs for the newly added volumes. All scenarios were then evaluated under varied slope values to estimate the influence of the slope value on the annual returns. Fig. 5 illustrates the comprehensive methodological framework used in this work.

**Table 2:** Summary of LNG revenue estimation scenarios.

	Scenario A.1	Scenario A.2	Scenario B.1	Scenario B.2
Selling Strategy	100% LTCs	100% LTCs	70% LTCs and 30% Spot	70% LTCs and 30% Spot
Pricing Mechanism	Brent-indexed	Brent-indexed for active contracts HH and TTF for new contracts	LTCs: Brent-indexed. Spot: TTF and JKM.	LTCs: Brent-indexed and HH Spot: TTF and JKM.
Baseline Slope	Brent: 11.79%	Brent: 11.79% HH: 115%	Brent: 11.79%	Brent: 11.79% HH: 115%
Uncontracted Volumes Market Share	N/A	Asia: 75% (HH) Europe: 25% (TTF)	Asia: 75% (HH) Europe: 25% (TTF)	Asia: 75% (HH) Europe: 25% (TTF)

For the state of Qatar, natural gas revenues contribute to around 65% of the hydrocarbon national GDP [127]. Consequently, forecasting natural gas revenues is crucial for fiscal planning and policymaking considering global transition efforts to renewables. LNG revenue forecast forms the basis of the country's cash flow plan, allowing a clear understanding of the state's current and future finances. Revenue forecasts allow governments to make informed decisions in different sectors, such as resource allocation, infrastructure development, education and healthcare, and taxation. However, the accuracy of the forecasts is subject to exogenous factors, including political influences, data precision, and the used forecasting approaches. In terms of the employed models, the availability of limited data points restricts testing and validating a selected model using a testing data set. Hence, statistical tests have been used to select the optimal model. Furthermore, the outcomes of this work have been compared with results published by prestigious international agencies to ensure consistency.

## 4 Results and discussion

The energy market has undergone several shocks in the last few years that restructured the market dynamics. As a major LNG exporter, Qatar, conversely, is entering a new era for LNG production and sale. From a supply perspective, the country is expanding its production capacity by more than 63% by 2026. From a demand perspective, the changes in contractual structures and the increased demand for LNG in European markets have made the exporting state reconsider its selling strategy. In today's market, tailoring to market needs is crucial to sustain sales of the added LNG volumes. This was supported by booking regasification terminals in the European market for spot selling and changing a few Asian contracts from JCC to brent indexation. Generally, a typical slope ranges between 10% and 16% of brent contracts [128]. Yet, the average slope value dropped from 13-14% to 10–11% in the past few years for Qatari SPAs [129]. In 2020, Qatar signed a minimum brent-indexed slope in the industry of 10.1%. In terms of pricing systems, Qatar has shifted toward including more gas hub-linked pricing, which considers the price of natural gas at significant trading hubs such as the UK NBP, the Dutch TTF, and the Platts JKM. However, the most economically attractive Qatari LNG selling approach in international markets remains unclear. Although LTCs are favourable to secure markets, spot sales allow the seller to seize opportunities and expand its market share. Hence, the outcomes of assessing different combinations of selling strategies are presented herein. The accuracy of revenue forecasts depends on the assumed average slope in contracts and the predicted prices. Consequently, the forecasted prices were compared with published forecasted prices by international agencies to assure consistency. A sensitivity assessment of the impact of slope variation on the overall revenue was conducted. Overall, quantitative models are beneficial for understanding the behaviour of forecasts. For example, assessments of different input parameters in exponential smoothing models support understanding the extent of historical prices' impact on forecasted values. This translates into supporting portfolio managers in making informed decisions on selling portfolio optimisation. A portfolio manager would be able to identify bullish markets and pricing systems and set the firm as a first mover in the energy market. In addition to opportunities to expand the market share, the advantage of the first mover marketing strategy supports the firm in the decision to introduce new energy products. This includes introducing greener energy commodities based on early market detections on shifts to renewable resources.

### 4.1 Price forecasting results

**Fig. 6:**
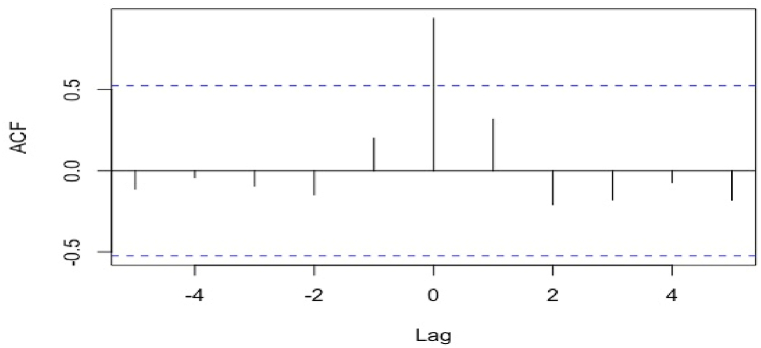
TTF-JKM pricing systems correlation at different time lags.

**Fig. 7:**
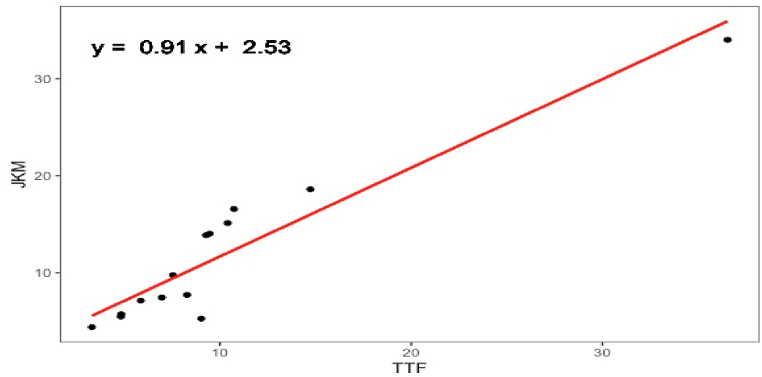
JKM-TTF linear regression analysis for average annual prices between 2009 and 2022.

In this work, four dominant pricing systems (i.e., brent, HH, TTF, and JKM) observed in active Qatari LNG contracts have been investigated qualitatively and quantitatively. Compared to studies in the literature that considered cyclic energy time series for short-term predictions, an annual time series with a frequency of 1 was investigated herein. Although brent, HH, and TTF pricing systems are mature, the JKM system, developed by S&P Global Platts, is a relatively new spot cargo benchmark launched in 2009. Hence, using around 13 average annual JKM prices is statistically infeasible to forecast a potential JKM trend until 2040. The TTF-JKM correlation was investigated to assess the degree of linkage. As illustrated in Fig. 6, the two pricing systems are highly correlated at lag = 0, with a correlation coefficient of 0.94. In contrast, no significant correlation is identified at other time lags less than or greater than 0. Additionally, a regression assessment of the TTF-JKM relationship revealed a strong relationship between the two pricing systems between 2009 and 2022. Statistical measures of R^2^ of 0.87 and a p-value <0.05 reflected the accuracy of the hypothesis. This justifies the validity of using the regression analysis to model future JKM values based on forecasted TTF values using the linear equation represented in Fig. 7. The correlation between the two pricing systems was also observed in an analysis of daily prices presented by Kpytin et al. [130]. It was further revealed that daily JKM-TTF correlation was impacted during COVID-19 pandemic where the two markets diverged

**Fig. 8:**
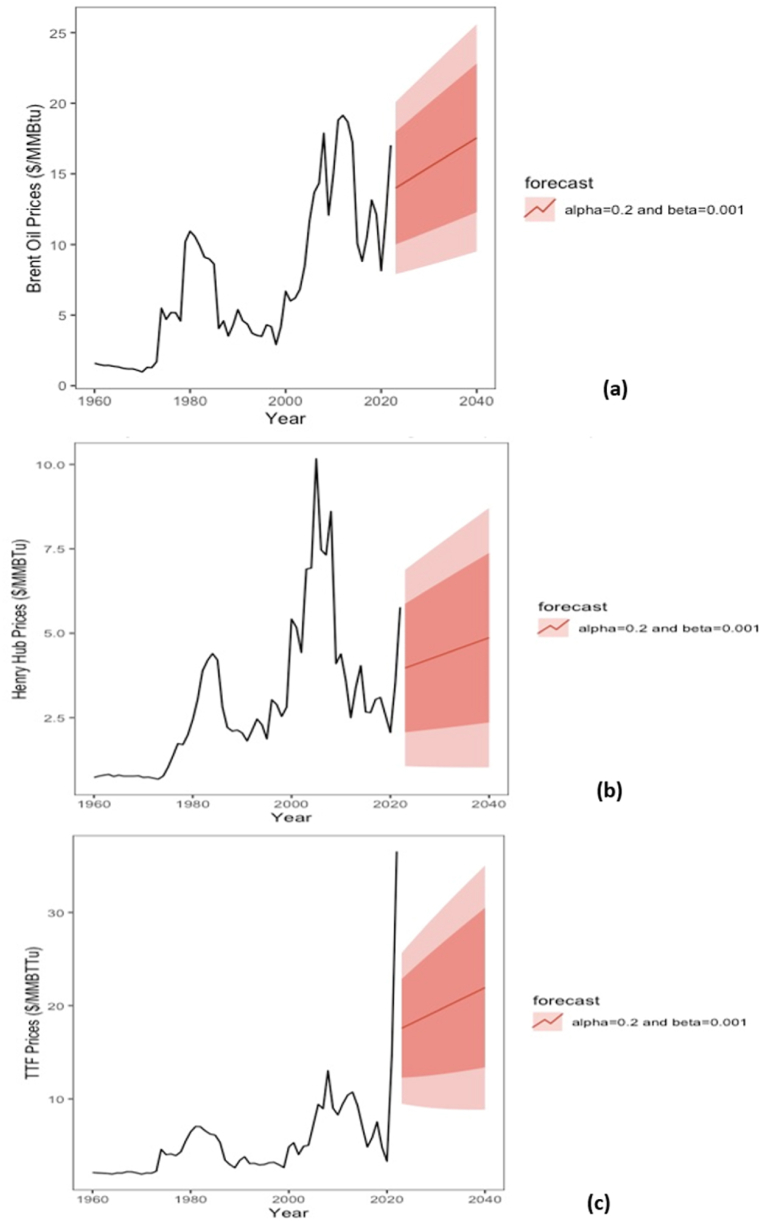
Forecasted brent, HH, and TTF prices between 2023 and 2040 using the double exponential smoothing (DES) model.

Given a few annual natural gas historical price data, identifying the underlying patterns in the time series data becomes challenging. Different classical models were examined, such as exponential smoothing (ES), autoregressive integrated moving average (ARIMA) and generalised autoregressive conditional heteroskedasticity (GARCH) models for price forecasting. Upon testing the models’ prerequisites (i.e., conditions to be met) prior to applying the model, some data failed to meet the requirements of ARIMA and GARCH. For example, ARIMA requires stationary data for reasonable forecasts. However, for the presented non-stationary data with trends and outliers, differencing was needed to convert a non-stationary time series into a differenced time series with constant mean and variance. Although differencing brent time series resulted in a stabilised returns time series, the auto.arima() function in R suggested an optimal ARIMA(0,0,0) model for fitting data and forecasting future outcomes. However, an ARIMA(0,0,0) model implies that the time series is a random walk with drift. This indicates that the future values of the time series are entirely unpredictable and can be forecasted as the last observed value plus a constant drift. This is because the ARIMA(0,0,0) model does not include any lagged values or seasonal components to account for the autocorrelation in the time series. Additionally, non-stationarity could not be met when differencing was applied to the HH and TTF time series, where randomness and noise in the data were increased. ES models were revealed as the core of the investigation due to the nonrequirement of stationary data. Since only annual data are considered to have a noticeable upward historical trend, DES was optimal to fit historical data and forecast future trends for brent, HH, and TTF pricing systems (Fig. 8). Table 3 summarises the characteristics and models for the pricing systems.

**Table 3:** Summary of characteristics and models used for the different pricing systems (brent, HH, and JKM).

	Brent	Henry Hub	Title Transfer Facility
Characteristic	Annual nonseasonal time series	Annual nonseasonal time series	Annual nonseasonal time series
Linearity (R^2^)	Mild 0.57	Poor 0.38	Poor 0.30
Trend	Stochastic trend	Stochastic trend	Stochastic trend
Stationarity (ADF test)	Not present	Not present	Not present
Trend Removal	Log returns differencing	Nonapplicable	Nonapplicable
ARIMA model	Partially Applicable	Strictly Nonapplicable	Strictly Nonapplicable
SES/DES model	Applicable	Applicable	Applicable

In data fitting using the DES model, α and β values are selected based on trial and error. More significant α and β values indicate more weight assignment to recent data and trends for forecasting future values. However, considering the market shocks between 2020 and 2022, assuming low reliance on the latest inflated prices is more reasonable for long-term forecasts. Lower α and β were investigated for forecasting future natural gas prices using the DES model. Fig. 10 illustrates the forecasted prices with an upward trend for each pricing system with 80% and 95% confidence intervals. The upper limits of the confidence intervals could represent a better market performance due to the higher unmeasured influence of tightened LNG supplies. On the contrary, the lower limits could reflect the market in case further LNG volumes are pumped into the global market. While the results of DES forecasts were selected due to the reasonability of the model fitting, statistical measures were further used to ensure the feasibility of DES over SES model. Table 4 summarises the statistical tests to compare the accuracy of the forecasts using DES vs SES for each pricing system. Fundamentally, lower MAPE, MAE, RMSE, AIC, and BIC are preferred for better fitting. However, achieving a lower value for the five statistical tests is nearly impossible. Overall, DES's upward projected price trend is an anticipated outcome driven by the implications of the Russian-Ukraine crisis on the global market, as reported by Prohorovs [131] and Hosoe [132]. Hence, the forecasted results were used to estimate the sales revenues, as discussed in the upcoming subsection.

**Table 4:** Statistical assessment for brent, HH, and TTF price forecasts using Simple Exponential Model and Double Exponential models.

	Brent		Henry Hub		Title Transfer Facility	
	SES α=0.2	DES α=0.2& β=0.001	SES α=0.2	DES α=0.2& β=0.001	SES α=0.2	DES α=0.3& β=0.001
**MAPE**	30.08	40.84	28.65	33.83	25.32	24.94
**MAE**	2.26	2.28	1.01	1.03	1.39	1.25
**RMSE**	3.14	3.01	1.45	1.43	2.11	1.953
**AIC**	409.29	405.87	311.73	312.8	352.49	344.90
**BIC**	413.57	412.30	316.02	319.15	356.74	351.29

### 4.2 Validation of forecasted prices

The latest geopolitical crisis between Russia and Ukraine has forever reshaped global energy markets. With accelerated efforts to transition to renewable energy in Europe, LNG will be a significant bridge fuel in Europe's mid-term and China's long-term markets. The shifts motivated several agencies to conduct qualitative and quantitative assessments of future natural gas market dynamics. Shell forecasted LNG demand to reach 650 to over 700 MTPA by 2040 [133]. Additionally, it suggested that more investments in liquefaction projects are needed to avoid an anticipated supply-demand gap in the late 2020s [133]. In the case of tight supplies, a business-as-usual scenario presented herein assumed a relative increase in the trend of natural gas prices until 2040. This upward trend is noticeable in quantitative brent, HH, and TTF trend forecasts using the DES model. The projections indicated a forecasted brent price of $81/bbl and $82/bbl in 2023 and 2024, respectively. This is compared to forecasted prices of $85/bbl in 2023 and $81/bbl in 2024, as reported by the EIA report released in April 2023 [134]. On the contrary, forecasts by Fitch were more bullish, with an average forecasted brent price of $90/bbl in 2023, falling to $83/bbl in 2024 [135]. While long-term forecasts in the field are uncommon due to the significant uncertainty involved, this analysis predicted an average brent price of $101.84/bbl in 2040. EIA reported a long-term projected average brent forecasted price of around $136/bbl as a reference case and $73/bbl as a low oil gas price [136]. At the same time, Deloitte predicted an average brent price of $103.2/bbl in 2040 in a report published in December 2022 [137]. Hence, the long-term projection for brent price is only 1.34% less than that predicted by Deloitte, with a difference of $1.36/bbl between the projected values.

For HH prices, average values of $3.97/MMBtu and $4.02/MMBtu were forecasted in this work for 2023 and 2024, respectively. This aligns relatively with EIA's projected prices of $3.40/MMBtu in 2023 and $4.04/MMBtu in 2024, reported earlier this year [138]. On the contrary, Deloitte reported bullish HH price forecasts of $5.30/MMBtu in 2023 and $4.82/MMBtu in 2024 [137]. The firm reported an anticipated long-term price of $4.34/MMBtu by 2040 compared to a predicted price of $4.87/MMBtu for the same year presented in this analysis. Conversely, more divergence was observed in the published projections for TTF price forecasts. Fitch Ratings predicted a bullish average TTF price of $19.29/MMBtu in 2023 and $9.64/MMBtu in 2024 [121]. The forecasted values are higher than the projected prices presented in this work. DES-based forecasts projected an average TTF spot price of $8.67/MMBtu and $8.78/MMBtu in 2023 and 2024, respectively. Fitch Ratings anticipated a significant drop of 50% in TTF prices by 2025, supported by aspirations for market rebalance after the Russian supply cut [121]. In the long term, more uncertainty is associated with the TTF pricing system, driven by possible new regulations. This includes the price corridor policy for indexing TTF price to other EU gas trading hubs, setting a benchmark price for LNG imports to the EU, and the recently approved price cap policy at the TTF trading point [139]. Hence, long-term projections of the TTF pricing system have been uncommon, unlike HH and brent pricing systems. Table 5 summarises the main findings of forecasted prices.

**Table 5:** A summary of forecasted prices compared with findings of other studies.

	Brent ($/bbl)		Henry Hub ($/MMBtu)		Title Transfer Facility ($/MMBtu)	
	**Current work**	**Other studies** [130,133]	**Current work**	**Other studies** [133,134]	**Current work**	**Other studies** [121]
**Selected model**	DES	N/A	DES	N/A	DES	N/A
**Forecasted price (2023)**	81	85	3.97	3.40	8.67	19.29
**Forecasted price (2024)**	82	81	4.02	4.04	8.78	9.64
**Forecasted price (2040)**	101.84	103.2	4.87	4.34	10.55	N/A

### 4.3 Revenue estimation results

The annual forecasted prices were then used to estimate Qatari LNG revenues until 2040 based on a scenario-based approach. As presented earlier in the methodology section, four scenarios comprising different potential selling strategies and pricing systems were considered:

1. Scenario A.1: 100% of the produced capacity is long-term contracted to brent crude.
2. Scenario A.2: All active contracted volumes are brent-indexed, while the new contracts are gas-indexed (HH and TTF)
3. Scenario B.1: 70% of the produced capacity is contracted based on brent crude indexation, while the remaining is sold in the spot market.
4. Scenario B.2: 70% of the produced capacity is contracted based on oil and gas indexation (brent, TTF, and HH), while the remaining is sold in the spot market (TTF and JKM).

**Fig. 9:**
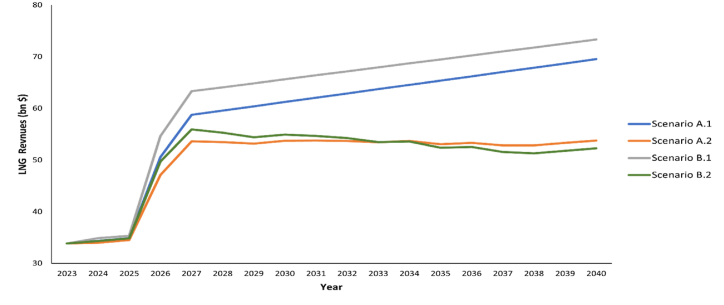
Estimated annual LNG revenues ($ bn) between (2023–2040) at different selling scenarios.

Due to the low production costs of Qatari natural gas, selling prices could always represent a win-win scenario for Qatar. Yet, the increased market competitiveness induced by high spot volumes from the flexible Australian and U.S. sellers complicates securing markets for Qatari LNG. Both sellers contributed to changing the market dynamics through flexible contracting and switching to gas-indexed pricing. As represented in Fig. 9, the revenues of pricing strategies have moved in the same direction since the start of the forecasts. The annual revenues only start to diverge from 2026 when new uncontracted volumes are pumped into the market. For Qatari LNG, scenario A.1 represents a classical approach to guarantee LNG sales and hedge against the volatility of natural gas prices. On the contrary, with increased interest in shifting to gas-indexation, scenario A.2 represents a potential selling approach where all newly signed SPAs are gas-indexed. Fundamentally, the latter approach is bullish for buyers and reflects a gradual diversion from oil-indexation to gas-indexation. The annual revenue estimation for both strategies justified that full reliance on oil indexation is preferable to the seller due to the higher premiums associated with oil indexations. A higher average annual revenue value of $58 bn is more economically attractive when considering brent-indexation in LTCs compared to an estimated average yearly revues value of $50 bn based on hybrid pricing systems strategy (i.e., brent, HH, and TTF) for LTCs.

**Fig. 10:**
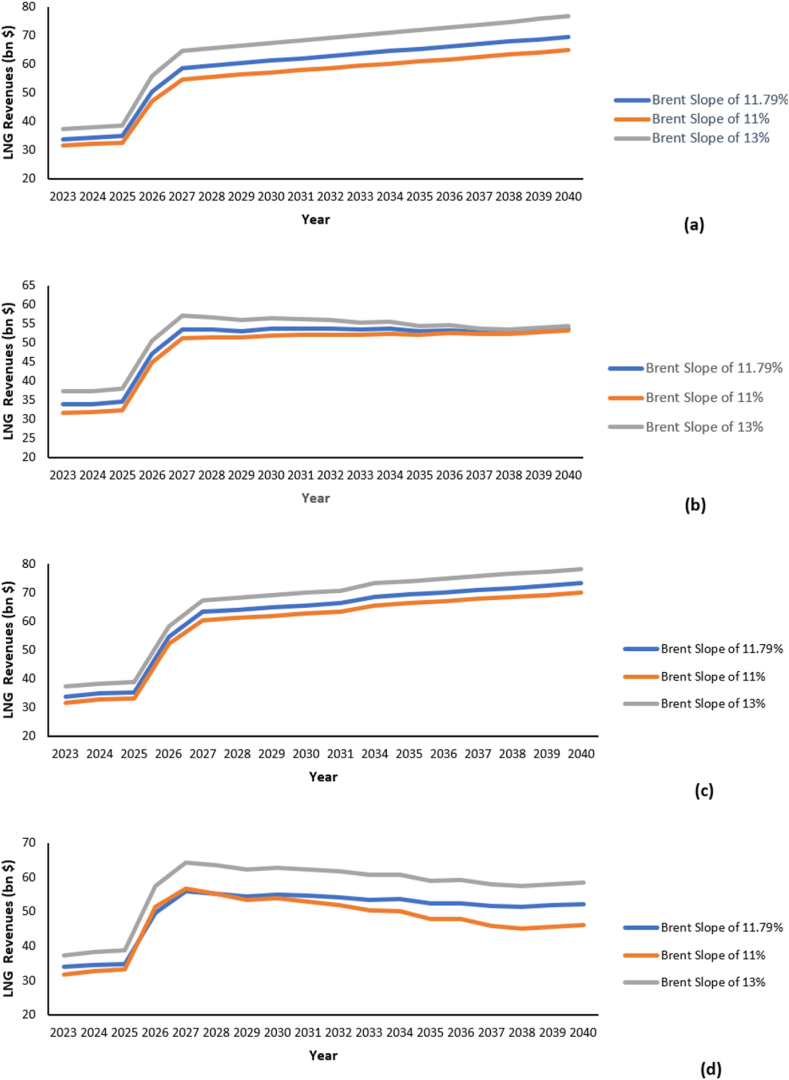
LNG revenues at different brent slops (11,11.79, and 13%) for (a) scenario A.1, (b) scenario A.2, (c) scenario B.1, and (d) scenario B.2.

Currently, Qatar aims to contract more than 90% of its production capacity on a long-term basis. With increased challenges in contracting the added volumes, scenarios B.1 and B.2 suggest reducing the share of LTCs to 70%. Interestingly, reducing the share of brent-indexed contracts to 70% in scenario B.1 resulted in the highest annual revenues, with an average forecasted annual revenue of $62 bn, as represented in Fig. 10. This was mainly supported by the 30% spot sales based on TTF and JKM indexations that offer higher premiums. On the contrary, shifting to HH indexation in the newly signed LTCs, as proposed in scenario B.2, lowers the average revenue value to $50 bn. Interestingly, the trend of revenues in scenarios A.1 and B.1 was observed to move in the same direction until 2040 despite the difference in selling strategies and the combination of selling prices. This is supported by the dominance of the brent-indexation in both selling strategies and its influence in securing higher premiums. Yet, minimising the brent-indexed contracts to 70% contributed significantly to enhancing the annual revenue. On the contrary, the year 2033 represents a turning point in the revenue estimates for scenarios A.2 and B.2. With many contracts expiring and increased availability of uncontracted LNG volumes, scenario B.2 provides lower premiums for gas-indexed LTCs compared to brent-indexed contracts. Overall, a diversified selling strategy was empirically proven more economically attractive, as suggested in former Qatar-based assessments by Meza & Koç [28] and Yusuf et al. [140,141].

In practice, LNG revenues are only published after finalising the sale of an LNG spot cargo or estimated upon signing a new contract. Hence, publishing forecasted aggregated LNG revenues is uncommon due to data and contract confidentiality. This, in turn, complicates comparing the outcomes of the revenue estimates presented herein with other studies. This analysis observed that 100% of the produced Qatari LNG is contracted for the current year. This, in turn, reduces uncertainty around the deviations between the used pricing systems. Compared to a total LNG achieved revenue of $18.3 bn (2020) and $29.6 bn (2021) [142], a revenue of $33.86 bn in 2023 was projected due to the surge in brent prices. On the contrary, with a few LTCs ending in the current year, buyers are yet to confirm contract renewal. In case no extensions are confirmed, the contracted LNG share will decline to 95% in 2024 and 2025 until the start of operations of the new LNG trains and contracts in 2026. Hence, with the increased share of uncontracted volumes, long-term revenue estimates possess uncertainties induced by pricing strategies, regional market shares, and pricing system variations. In this work, a business-as-usual scenario predicted higher LNG volumes to be absorbed by the Asian markets based on brent-indexed LTCs at slope values of 10–13%. Considering the shipping costs, our analysis concludes that scenarios A.1 and B.1 reflect the most probable strategy for LNG exports from Ras Laffan to the Asian markets. At the same time, LNG volumes produced from Sabine Pass in the U.S. could potentially be dedicated to mid-term and spot sales in the European markets based on HH and TTF indexation. However, this assessment is solely concerned with the LNG volumes produced and shipped from Ras Laffan.

### 4.4 Revenue estimation sensitivity analysis

In addition to price forecast accuracy, several factors influence the estimated annual revenues, including slope value, change in contractual structures and market share. In a business-as-usual scenario of fixed price forecasts and market dynamics, a sensitivity assessment was conducted to quantify the influence of slope variation on the estimated revenues. Announced brent slopes used in Qatari LTCs have typically ranged between 10.19 and 13.37%. Hence, slope values of 11%, 11.79%, and 13% were used to quantify the degree of indexation change on the revenues, as represented in Fig. 10. Results demonstrate that the slope value is directly proportional to the revenues, wherein higher slopes result in enhanced revenues. In practice, brent slopes tend to have an upward ceiling of 17.2%. Hence, slopes lower than the upward ceiling indicate that LNG is being discounted to brent subject to the same caloric value. Moreover, Qatar is anticipated to maintain lower slopes to sustain long-term buyers. Based on market performance, it could be common to witness slope negotiations of active contracts in the following years. In practice, slopes are renegotiated every 4–5 years to meet the mutual interests of buyers and sellers. Consequently, the selling portfolios are periodically reevaluated on (1) a quarterly basis to accommodate changes in commodities prices quarterly or (2) annually to account for slope negotiations.

## 5 Policy and portfolio implications

From a policy implication perspective, the findings provide valuable insights for decision-makers and policymakers. Our findings reveal that the lack of liquefaction capacity investments in the last few years has implications for a tight supply until 2040. This supports an upward trend for predicted annual natural gas prices. Policymakers should carefully consider liquefaction financing to avoid tight supply situations that could hinder energy security. When accounting for social responsibility, elevated prices jeopardise the access of price-sensitive buyers to cleaner energy resources. While social responsibility is essential, policymakers should balance social responsibility and profitability. Similarly, in alignment with environmental sustainability targets, long-term investments in renewables must be considered for a smooth transition to renewables beyond 2050. The state of Qatar, the rich natural gas exporter, is anticipated to expand its LNG production by around 64% in the year 2026. The changes in global market dynamics in terms of shifts to spot sales, short-term contracts, and natural gas indexation presented challenges in signing contracts in the last few years. This, in turn, promotes investigating alternative selling strategies away from the classical long-term oil-indexed approach. The proposed scenarios (summarised in Table 6) reported annual revenue estimates based on different selling strategies (i.e., LTCs and spot selling) and price indexations (i.e., brent, HH, TTF, and JKM). For Qatari LNG, oil-indexed LTCs have been favoured for contracting more than 90% of the production capacity. However, the results reveal that reducing the long-term contracted oil-indexed LNG volumes to 70% and dedicating the remaining capacities to spot gas-indexed sales is economically attractive. Interestingly, higher premiums associated with TTF and JKM pricing were revealed to boost the revenues. On the contrary, shifting from brent-indexation to HH-indexation in LTCs resulted in lower LNG premiums. The selling strategies assessment reveals that sustaining 70% brent-indexed LTCs could be achieved when selling in different regional markets. The remaining 30% of uncontracted volumes could be dedicated to spot selling in European and Asian markets based on TTF and JKM pricing systems, respectively. This, in turn, will support Qatar in expanding its spot market share to meet the seasonal demand of buyers and utilise high market opportunities.

**Table 6:** A summary of estimated average annual revenues for Qatari LNG sales in international markets based on four selling strategies.

	Scenario A.1	Scenario A.2	Scenario B.1	Scenario B.2
**Selling Strategy**	100% LTCs	100% LTCs	70% LTCs and 30% Spot	70% LTCs and 30% Spot
**Pricing Mechanism**	Brent-indexed	Brent-indexed for active contracts HH and TTF for new contracts	LTCs: Brent-indexed. Spot: TTF and JKM.	LTCs: Brent-indexed and HH Spot: TTF and JKM.
**Average annual revenue**	$58 bn	$50 bn	$62 bn	$50 bn

## 6 Conclusion

Several factors have contributed to restructuring energy markets driven by energy security and environmental sustainability targets in the last few years. The latest shocks in the last decade (i.e., the 2015 oil crisis, the COVID-19 pandemic, and the Russian-Ukraine war) have vigorously accelerated the efforts to diversify energy sources and importing portfolios. Natural gas has been considered the cleanest fossil fuel and is widely accepted as a transition fuel to renewables. However, the latest disturbances have significantly impacted different parts of natural gas supply chains, from project financing to securing long-term buyers for existing projects. The latter was mainly induced by buyers' preferences to shift from long-term oil-indexed contracts to short-term and spot gas-indexed deals. For oil and gas economies, including Qatar, demand insecurity threatens the economic sustainability of oil and gas businesses. This work developed a predictive-perspective framework to assess different selling strategies due to market restructurings empirically. Using historical natural gas price data and contract data as input, a three-step methodology was developed. First, future brent, HH, TTF, and JKM prices were forecasted using the double exponential smoothing (DES) model. The forecasted prices and contract data were then used to estimate annual Qatari LNG revenue between 2023 and 2040 based on four selling scenarios. The selling strategies were categorised into two main strategies: (A) 100% long-term contracts, and (B) 70% long-term contracts and 30% spot sale strategy. Combinations of different pricing systems (i.e., brent, HH, TTF, and JKM) were employed in each category. Lastly, a sensitivity analysis on the selling strategies was conducted to assess the influence of changing the brent-slope on the calculated revenue. The empirical tools utilised in the methodological framework yielded interesting outcomes. In a business-as-usual scenario where no further liquefaction investments are anticipated in the near future, an upward trend of future brent and natural gas prices is expected until 2040. This was revealed by the forecasts given by the double exponential smoothing (DES) model for the different pricing systems. Brent was observed to be the most dominant pricing system in the Qatari contracts. The DES model predicted a brent price of $81/bbl in 2023 and $82/bbl in 2024. This is compared to EIA's forecasts of $85/bbl and $81/bbl in 2023 and 2024, respectively. Additionally, the scenario-based analysis for annual revenue estimate revealed that brent indexation is more attractive than hybrid pricing for long-term contracts. For revenue estimates between 2023 and 2040, brent indexation with a 17.86% slope resulted in an average revenue of $58.42 billion, around 15% greater than the average annual revenue estimated based on mixed indexations. On the contrary, reducing the volumes dedicated to long-term contracts to 70% and allocating the remaining capacities to spot-indexation enhanced the overall annual revenue. In this scenario, contracting long-term volumes based on brent-indexation and selling the remaining spot capacities based on hybrid gas indexations (i.e., TTF and JKM) resulted in an average annual revenue of $62 billion. Projections of HH prices reflected that HH is the most liquid gas pricing system. However, HH with a 115% slope yields lower premiums for sellers. This justifies the position of Qatari sellers to rely on other indexations for pricing contracted LNG volumes. Consequently, only higher HH-indexation slope values would be economically justifiable for Qatar to substitute long-term brent-indexation with HH-indexation. The outcomes of the analysis support decision-makers and policymakers in understanding the implications of the latest market structures on the sellers and buyers. For markets, the lack of investment in new liquefaction capacities will intensify an upward trend of prices until 2040. From a buyers' perspective, diversifying selling strategies and increasing the share of spot selling could lead to higher premiums and market share.

In terms of the limitations, the study relies heavily on historical data. It may not fully capture the complexities of future crises and their unique impacts on the natural gas market. Additionally, forecasting future prices inherently involves uncertainties arising from unforeseen events or exogenous shocks. An important limitation of the existing study is the model's capacity to adequately capture uncertainty within the natural gas market dynamics, particularly during periods of crisis. The selection of more advanced models to consider uncertainties is subject to data availability, which is applicable to daily price forecasts. As a result, the projections generated by the model should be interpreted with caution, recognising the potential for unforeseen events or abrupt shifts in market conditions to deviate from forecasted outcomes.

As this study considered a business-as-usual scenario for predicting future market dynamics, future studies should include multi-price scenarios based on potential decarbonisation strategies. Multi-price scenarios would support decision-makers in the strategic planning of production capacities. Additionally, uncertainty analysis could be incorporated through using models to consider the stochasticity of forecasted prices. In terms of investigating the optimal selling strategy for uncontracted LNG volumes, portfolio managers should consider robust optimisation models to maximise LNG profitability using stochastic forecasts. Finally, modelling LNG producers' interactions in different regional markets would support portfolio managers in allocating LNG volumes based on market needs.

## CRediT authorship contribution statement

**Noor Yusuf:** Conceptualization, Writing – original draft, Visualization, Validation, Software, Methodology, Investigation, Formal analysis, Data curation. **Rajesh Govindan:** Conceptualization, Methdology, Writing – review & editing, Validation, Investigation, Data curation. **Tareq Al-Ansari:** Conceptualization, Methodology, Writing – review & editing, Supervision, Project administration, Resources.

## Declaration of competing interest

The authors declare that they have no known competing financial interests or personal relationships that could have appeared to influence the work reported in this paper.
